# A practical approach to phylogenomics: the phylogeny of ray-finned fish (Actinopterygii) as a case study

**DOI:** 10.1186/1471-2148-7-44

**Published:** 2007-03-20

**Authors:** Chenhong Li, Guillermo Ortí, Gong Zhang, Guoqing Lu

**Affiliations:** 1 School of Biological Sciences, University of Nebraska, Lincoln, NE 68588, USA; 2 Department of Mathematics, University of Nebraska, Omaha, NE 68182, USA; 3 Department of Biology, University of Nebraska, Omaha, NE 68182, USA

## Abstract

**Background:**

Molecular systematics occupies one of the central stages in biology in the genomic era, ushered in by unprecedented progress in DNA technology. The inference of organismal phylogeny is now based on many independent genetic loci, a widely accepted approach to assemble the tree of life. Surprisingly, this approach is hindered by lack of appropriate nuclear gene markers for many taxonomic groups especially at high taxonomic level, partially due to the lack of tools for efficiently developing new phylogenetic makers. We report here a genome-comparison strategy to identifying nuclear gene markers for phylogenetic inference and apply it to the ray-finned fishes – the largest vertebrate clade in need of phylogenetic resolution.

**Results:**

A total of 154 candidate molecular markers – relatively well conserved, putatively single-copy gene fragments with long, uninterrupted exons – were obtained by comparing whole genome sequences of two model organisms, *Danio rerio *and *Takifugu rubripes*. Experimental tests of 15 of these (randomly picked) markers on 36 taxa (representing two-thirds of the ray-finned fish orders) demonstrate the feasibility of amplifying by PCR and directly sequencing most of these candidates from whole genomic DNA in a vast diversity of fish species. Preliminary phylogenetic analyses of sequence data obtained for 14 taxa and 10 markers (total of 7,872 bp for each species) are encouraging, suggesting that the markers obtained will make significant contributions to future fish phylogenetic studies.

**Conclusion:**

We present a practical approach that systematically compares whole genome sequences to identify single-copy nuclear gene markers for inferring phylogeny. Our method is an improvement over traditional approaches (e.g., manually picking genes for testing) because it uses genomic information and automates the process to identify large numbers of candidate makers. This approach is shown here to be successful for fishes, but also could be applied to other groups of organisms for which two or more complete genome sequences exist, which has important implications for assembling the tree of life.

## Background

The ultimate goal of obtaining a well-supported and accurate representation of the tree of life relies on the assembly of phylogenomic data sets for large numbers of taxa [1]. Molecular phylogenies based on DNA sequences of a single locus or a few loci often suffer from low resolution and marginal statistical support due to limited character sampling. Individual gene genealogies also may differ from each other and from the organismal phylogeny (the "gene-tree vs. species-tree" issue) [2,3], in many cases due to systematic biases (i.e., compositional bias, long-branch attraction, heterotachy), leading to statistical inconsistency in phylogenetic reconstruction [4-7]. Phylogenomic data sets – using genome sequences to study evolutionary relationship – provide the best solution to these problems [1,8]. This approach requires compilation of large data sets that include many independent nuclear loci for many species [9-14]. Such data sets are less likely to succumb to sampling and systematic errors [13] by offering the possibility of analyzing large numbers of phylogenetically informative characters from different genomic locations, and also of corroborating phylogenetic results by varying the species sampled. If any systematic bias may be present in a fraction of individual loci sampled, it is unlikely that all affected loci will be biased in the same direction. Powerful analytical approaches that accommodate model heterogeneity among data partitions are becoming available to efficiently analyze such complex phylogenomic data sets [15,16].

Constructing phylogenomic data sets for large number of taxa still is, however, quite challenging. Most attempts to use this approach have been based either on few available complete genomic sequence data [13,17,18], or cDNA and ESTs sequences [9,12,18,19] for relatively few taxa. Availability of complete genomes limits the number of taxa that can be analyzed [13,17], imposing known problems for phylogenetic inference associated with poor taxon sampling [20,21]. On the other hand, methods based on ESTs or cDNA sequence data are not practical for many taxa because they require construction of cDNA libraries and fresh tissue samples. In addition, some genes may not be expressed in certain tissues or developmental stages, leading to cases with undesirable amounts of missing data [9]. The most efficient way to collect nuclear gene sequences for many taxa is to directly amplify target sequences using "universal" PCR primers, an approach so far used for just a few widely-used nuclear genes [22-25], or selected taxonomic groups (e.g., placental mammals and land plants). Widespread use of this strategy in most taxonomic groups has been hindered by the paucity of available PCR-targeted gene markers.

**Figure 1:**
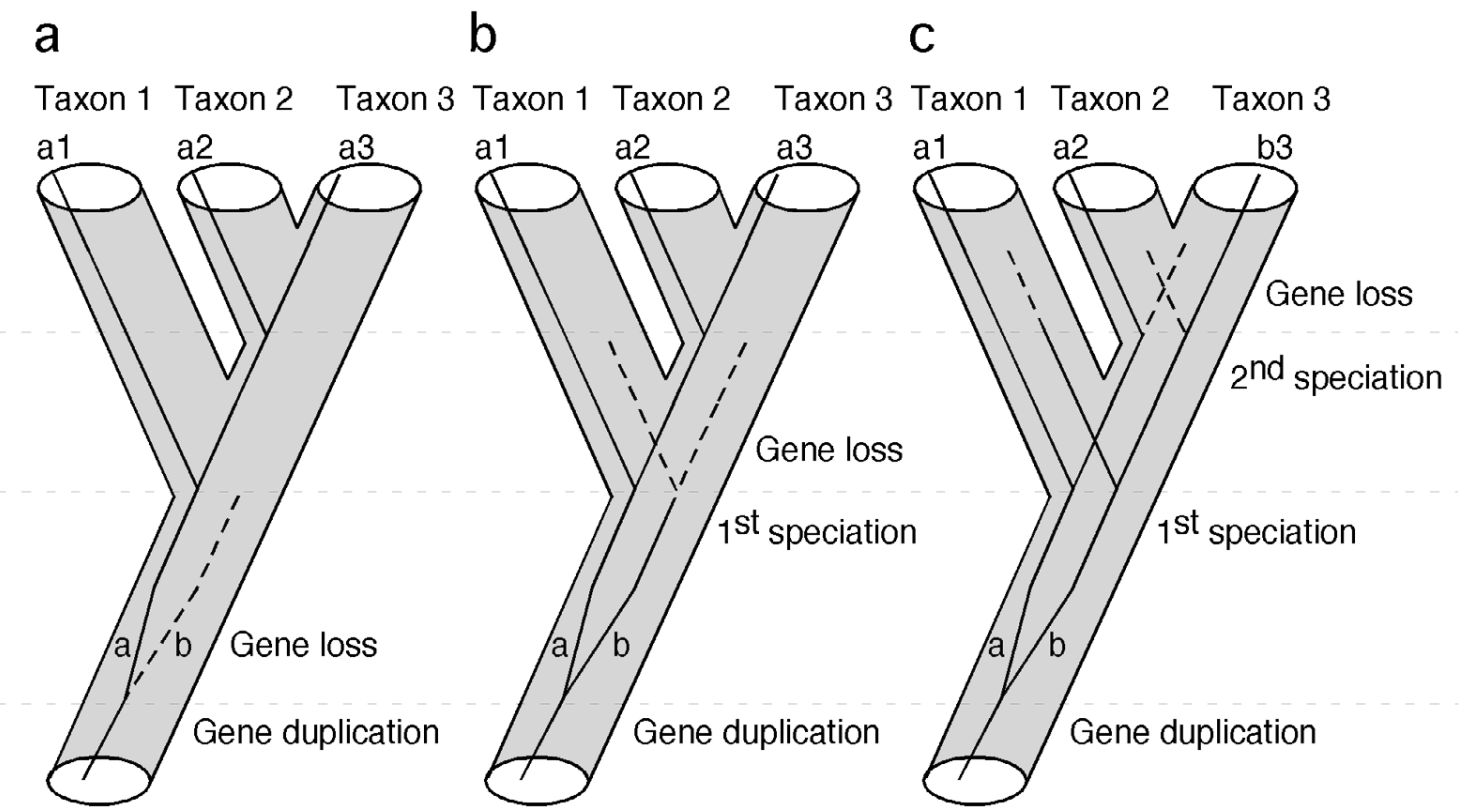
**Single-copy genes are useful markers for phylogeny inference**. Gene duplication and subsequent loss may not cause incongruence between gene tree and species tree if gene loss occurs before the first speciation event (a), or before the second speciation event (b). The only case that would cause incongruence is when the gene survived both speciation events and is asymmetrically lost in taxon 2 and taxon 3 (c).

Mining genomic data to obtain candidate phylogenetic markers requires stringent criteria, since not all loci are likely to carry the appropriate historical signal. The phylogenetic informativeness of characters has been extensively debated on theoretical grounds [26,27], as well as in empirical cases [28-30]. Our study does not intend to contribute to this debate, but rather to focus on the practical issues involved in obtaining the raw data for analysis. What is the best strategy to select a few hundreds candidate loci from thousands of genes present in the genome? For practical purposes, a good phylogenetic nuclear gene marker must satisfy three criteria. First, orthologous genes should be easy to identify and amplify in all taxa of interest. One of the main problems associated with nuclear protein-coding genes used to infer phylogeny is uncertainty about their orthology [3]. This is especially true when multiple copies of a target gene are amplified by PCR from whole genomic DNA. To minimize the chance of sampling paralogous genes among taxa (the trap of "mistaken paralogy" that will lead to gene-tree-species-tree discordance), our approach is initiated by searches for single-copy nuclear genes in genomic databases. Under this criterion, even if gene duplication events may have occurred during evolution of the taxa of interest (e.g., the fish-specific whole-genome duplication event) [31,32], duplicated copies of a single-copy nuclear gene tend to be lost quickly, possibly due to dosage compensation [33]. Some authors estimate that almost 80% of the paralogs have been secondarily lost following the genome-duplication event [34,35]. Thus, if duplicated copies are lost before the relevant speciation events occur (Figure 1a, b), no paralogous gene copies would be sampled. If the alternative situation occurs (Figure 1c), paralogy will mislead phylogenetic inference resulting in topological discordance among genes. In the latter case, the topological distribution of this discordance may be used to reconstruct putative duplication/extinction events and clarify the putative mistaken paralogy [36]. The second criterion used to facilitate efficient data collection is to identify protein-coding genes with long exons (longer than a practical threshold determined by current DNA sequencing technology, for example 800 bp). Most genes are fragmented into small exons and large introns. For high taxonomic-level phylogenetic inference (deep phylogeny), intron sequences evolve too fast and are usually not informative, becoming an obstacle for the amplification and sequencing of more informative exon-coding sequences. The third criterion used seeks to identify reasonably conserved genes. Genes with low rates of evolution are less prone to accumulate homoplasy, and also provide the practical advantage of facilitating the design of universal primers for PCR that will work on a diversity of taxa. Furthermore, conserved protein-coding genes also are easy to align for analysis, based on their amino acid sequence.

Sequence conservatism and long exonic regions have been used as preferred criteria to select phylogenetic markers in the past [37]. However, finding many preferred, easy-to-apply gene markers is unlikely when candidate genes are manually screened from data bases or taken from isolated studies of few individual genes. This complexity partially explains the scarcity of currently available nuclear gene markers in many taxonomic groups. To address the problem, we present a simple bioinformatic approach to obtain nuclear gene markers from complete genomic data, based on the three aforementioned criteria. Our method incorporates two improvements over the traditional way of manually picking genes and testing their phylogenetic utilities. These improvements include using full genomic information and automating the process of searching for candidate makers. We apply the method to Actinoptertygii (ray-finned fish), the largest vertebrate clade – they make up about half of all known vertebrate species – with a poorly known phylogeny [38-42]. We also present experimental tests to show that PCR primers designed for a subset of the candidate markers can efficiently amplify these markers for a highly diverse sample of ray-finned fishes. Comparative analyses of the sequences obtained show encouraging phylogenetic properties for future studies.

## Results

**Figure 2:**
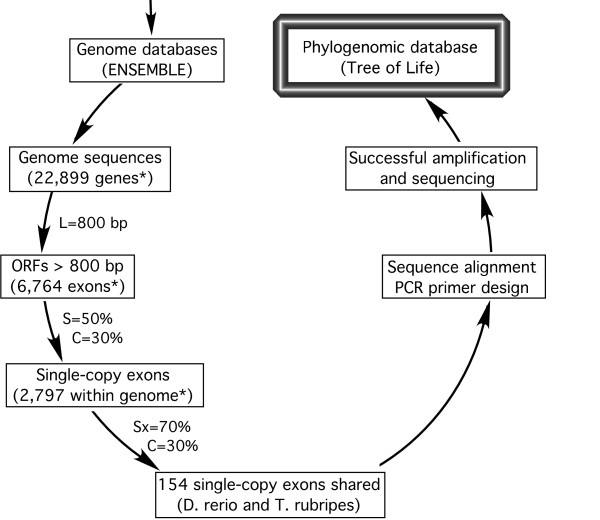
**The bioinformatic pipeline for phylogenetic markers development**. It involves within- and across-genome sequences comparison, *in silico *test with sequences in other species, and experimental validation. Numbers of genes and exons identified for *D. rerio *are indicated by the asterisk. Exon length (L), within-genome similarity (S), between-genome similarity (Sx), and coverage (C) are adjustable parameters (see methods).

The bioinformatic pipeline used is shown in Figure 2. Within-genome sequence comparisons resulted in 2,797 putative single-copy exons (> 800 bp) in zebrafish (*D. rerio*), and 1,822 in torafugu (*T. rubripes*), 2132 in stickleback (*G. aculeatus*), and 1809 in Japanese rice fish (*O. latipes*). Note that our operational definition of a "single-copy" gene only requires that the fragment is not present as a second copy in the genome with similarity higher than 50%. Some single-copy genes may, in fact, have duplicates in the genome that are less than 50% similar. Pairwise between-genome comparisons of the single-copy exon sequences resulted in a range of 113 to 281 putative orthologs shared among genomes, that have similarity greater than 70%. The lowest number of "conserved orthologs" was detected between zebrafish and rice fish, and the highest between torafugu and stickleback. The number of putative conserved orthologs shared among three or more genomes varied from case to case; for example, it peaked at 155 when comparing torafugu, Japanese rice fish, and stickleback, but only 61 for the comparison involving torafugu, Japanese ricefish, and zebrafish. All the information resulting from these analyses is publicly available in our website [43], and a sample output of candidate markers is shown in Additional file 1.

To investigate the properties of candidate markers, we analyzed those found in the zebrafish and torafugu comparison, since their genome sequences are well annotated. Among them, 154 putative homologs were identified between zebrafish and torafugu by cross-genome comparison. Further comparison with EST sequences from other fish species reduced this number to 138 candidate markers (Supplementary Table 1). The 154 candidate markers shared between these two genomes according to our search criteria are distributed among 24 of the 25 chromosomes of zebrafish, and a Chi-square test did not reject a Poisson distribution of markers among chromosomes (χ^2 ^= 16.99, df = 10, p = 0.0746). The size of candidate markers ranged from 802 to 5811 bp (in *D. rerio*). Their GC content ranged from 41.6% to 63.9% (in *D. rerio*), and the average similarity of the DNA sequence of these markers between *D. rerio *and *T. rubripes *varied from 77.3% to 93.2% (constrained by the search criteria).

To test the practical value of potential phylogenetic markers, 15 gene fragments were randomly picked from the candidate list of 154 and tested experimentally on 36 taxa, chosen to represent two-thirds of all ray-finned fish orders (see Additional file 2). PCR primers were designed on conserved flanking regions for each fragment, based on the genomic sequences and tested on all taxa (Table 1). Ten out of the 15 markers examined successfully amplified a single product of the predicted size by a nested PCR approach in 31 taxa. For comparative sequence analyses, we took only 14 taxa (*Amia calva*, *D. rerio*, *Semotilus atromaculatus*, *Ictalurus punctatus*, *Oncorhynchus mykiss*, *Brotula multibarbata*, *Fundulus heteroclitus*, *Oryzias latipes*, *Oreochromis niloticus*, *Gasterosteus aculeatus*, *Lycodes atlanticus*, *T. rubripes*, *Morone chrysops*, *Lutjanus mahogoni*) that could be amplified and sequenced directly for the set of 10 markers [GenBank: EF032909 – EF033038]. The size of the sequenced fragments ranged from 666 to 987 bp, and the average uncorrected genetic distances for DNA sequence of the 10 markers among the 14 taxa ranged from 13% to 21%. We present (Table 2) additional characteristics of the data set such as the substitution rate, consistency index (CI), gamma shape parameter (α), relative composition variability (RCV), and treeness [44] resulting from phylogenetic analysis of the sequences of the 10 new markers. Values obtained are similar to those observed in a commonly used phylogenetic marker – recombination activating gene 1 (RAG-1, Table 2). For the newly characterized phylogenetic markers, the substitution rate is negatively correlated with CI (r = -0.84, P = 0.0026) and marginally correlated with α (r = -0.56, P = 0.095). In contrast, base composition heterogeneity (RCV) and the phylogenetic signal to noise index (treeness index) are not correlated with substitution rate. Based on the treeness value, genes ENC1, plagl2, Ptr, Gylt and tbr1 seem well suited for phylogenetic studies at high taxonomic level among ray-finned fishes.

**Table 1:** PCR primers and annealing temperatures used to amplify 10 new markers

Gene*	Primers	Sequences	Annealing temp	PCR steps
zic1	zic1_F9	5' GGACGCAGGACCGCARTAYC 3'	57	1st
	zic1_R967	5' CTGTGTGTGTCCTTTTGTGRATYTT 3'		PCR
	zic1_F16	5' GGACCGCAGTATCCCACYMT 3'	57	2nd
	zic1_R963	5' GTGTGTCCTTTTGTGAATTTTYAGRT 3'		PCR
myh6	myh6_F459	5' CATMTTYTCCATCTCAGATAATGC 3'	53	1st
	myh6_R1325	5' ATTCTCACCACCATCCAGTTGAA 3'		PCR
	myh6_F507	5' GGAGAATCARTCKGTGCTCATCA 3'	62	2nd
	myh6_R1322	5' CTCACCACCATCCAGTTGAACAT 3'		PCR
RYR3	RYR3_F15	5' GGAACTATYGGTAAGCARATGG 3'	55	1st
	RYR3_R968	5' TGGAAGAAKCCAAAKATGATGC 3'		PCR
	RYR3_F22	5' TCGGTAAGCARATGGTGGACA 3'	62	2nd
	RYR3_R931	5' AGAATCCRGTGAAGAGCATCCA 3'		PCR
Ptr	Ptr_F458	5' AGAATGGATWACCAACACYTACG 3'	55	1st
	Ptr_R1248	5' TAAGGCACAGGATTGAGATGCT 3'		PCR
	Ptr_F463	5' GGATAACCAACACYTACGTCAA 3'	62	2nd
	Ptr_R1242	5' ACAGGATTGAGATGCTGTCCA 3'		PCR
tbr1	tbr1_F1	5' TGTCTACACAGGCTGCGACAT 3'	57	1st
	tbr1_R820	5' GATGTCCTTRGWGCAGTTTTT 3'		PCR
	tbr1_F86	5' GCCATGMCTGGYTCTTTCCT 3'	62	2nd
	tbr1_R811	5' GGAGCAGTTTTTCTCRCATTC 3'		PCR
ENC1	ENC1_F85	5' GACATGCTGGAGTTTCAGGA 3'	53	1st
	ENC1_R982	5' ACTTGTTRGCMACTGGGTCAAA 3'		PCR
	ENC1_F88	5' ATGCTGGAGTTTCAGGACAT 3'	62	2nd
	ENC1_R975	5' AGCMACTGGGTCAAACTGCTC 3'		PCR
Gylt	Glyt_F559	5' GGACTGTCMAAGATGACCACMT 3'	55	1st
	Glyt_R1562	5' CCCAAGAGGTTCTTGTTRAAGAT 3'		PCR
	Glyt_F577	5' ACATGGTACCAGTATGGCTTTGT 3'	62	2nd
	Glyt_R1464	5' GTAAGGCATATASGTGTTCTCTCC 3'		PCR
SH3PX3	SH3PX3_F461	5' GTATGGTSGGCAGGAACYTGAA 3'	55	1st
	SH3PX3_R1303	5' CAAACAKCTCYCCGATGTTCTC 3'		PCR
	SH3PX3_F532	5' GACGTTCCCATGATGGCWAAAAT 3'	62	2nd
	SH3PX3_R1299	5' CATCTCYCCGATGTTCTCGTA 3'		PCR
plagl2	plagl2_F9	5' CCACACACTCYCCACAGAA 3'	55	1st
	plagl2_R930	5' TTCTCAAGCAGGTATGAGGTAGA 3'		PCR
	plagl2_F51	5' AAAAGATGTTTCACCGMAAAGA 3'	62	2nd
	plagl2_R920	5' GGTATGAGGTAGATCCSAGCTG 3'		PCR
sreb2	sreb2_F10	5' ATGGCGAACTAYAGCCATGC 3'	55	1st
	sreb2_R1094	5' CTGGATTTTCTGCAGTASAGGAG 3'		PCR
	sreb2_F27	5' TGCAGGGGACCACAMCAT 3'	62	2nd
	sreb2_R1082	5' CAGTASAGGAGCGTGGTGCT 3'		PCR

*Gene markers are named following annotations in ENSEMBLE. zic1, zic family member 1; myh6, myosin, heavy polypeptide 6; RYR3 (si:ch211-189g6.1), novel protein similar to vertebrate ryanodine receptor 3; Ptr (si:ch211-105n9.1), hypothetical protein LOC564097; tbr1, T-box brain 1; ENC1(559445 Entrezgene), similar to ectodermal-neural cortex 1; Glyt (zgc:112079), glycosyltransferase; SH3PX3, similar to SH3 and PX domain containing 3 gene; plagl2, pleiomorphic adenoma gene-like 2; sreb2, Super conserved receptor expressed in brain 2.

**Table 2:** Summary information of the 10 gene markers amplified in 14 taxa

Gene	Exon ID	No. of bp	No. of var.	No. of PI	Genetic distance (%)	Sub. rate	CI-MP	α	RCV	Treeness
zic1	ENSDARE00000015655	894	296	210	13(2.3–22.6)	0.64	0.61	1.64	0.13	0.23
myh6	ENSDARE00000025410	735	323	235	18(7.8–23.2)	1.35	0.54	0.68	0.11	0.22
RYR3	ENSDARE00000465292	825	389	258	18(8–23.6)	1.25	0.56	0.67	0.11	0.21
Ptr	ENSDARE00000145053	705	304	234	18(5.3–28.1)	1.03	0.57	1.64	0.12	0.29
tbr1	ENSDARE00000055502	666	256	170	14(3–25.6)	0.65	0.67	2.91	0.10	0.28
ENC1	ENSDARE00000367269	810	312	248	16(6.7–24.3)	1.13	0.55	1.10	0.16	0.33
Gylt	ENSDARE00000039808	870	463	335	21(6.6–29.7)	1.18	0.60	1.70	0.12	0.27
SH3PX3	ENSDARE00000117872	705	290	226	16(6.2–24)	1.11	0.55	1.53	0.14	0.22
plagl2	ENSDARE00000136964	675	250	184	14.3(5.1–21.5)	0.81	0.61	0.92	0.10	0.33
sreb2	ENSDARE00000029022	987	344	225	13(4–21.6)	0.85	0.61	0.88	0.11	0.23
RAG1	-	1344	684	514	20(8.1–29)	1.28	0.57	1.68	0.05	0.23

bp, base pairs; var., variable sites; PI, parsimony informative sites; Genetic distance, average uncorrected distance, number in parenthesis are range of the distances; Sub. rate, relative substitution rate estimated using Bayesian approach; CI-MP, consistency index; α, gamma distribution shape parameter; RCV, relative composition variability.

**Figure 3:**
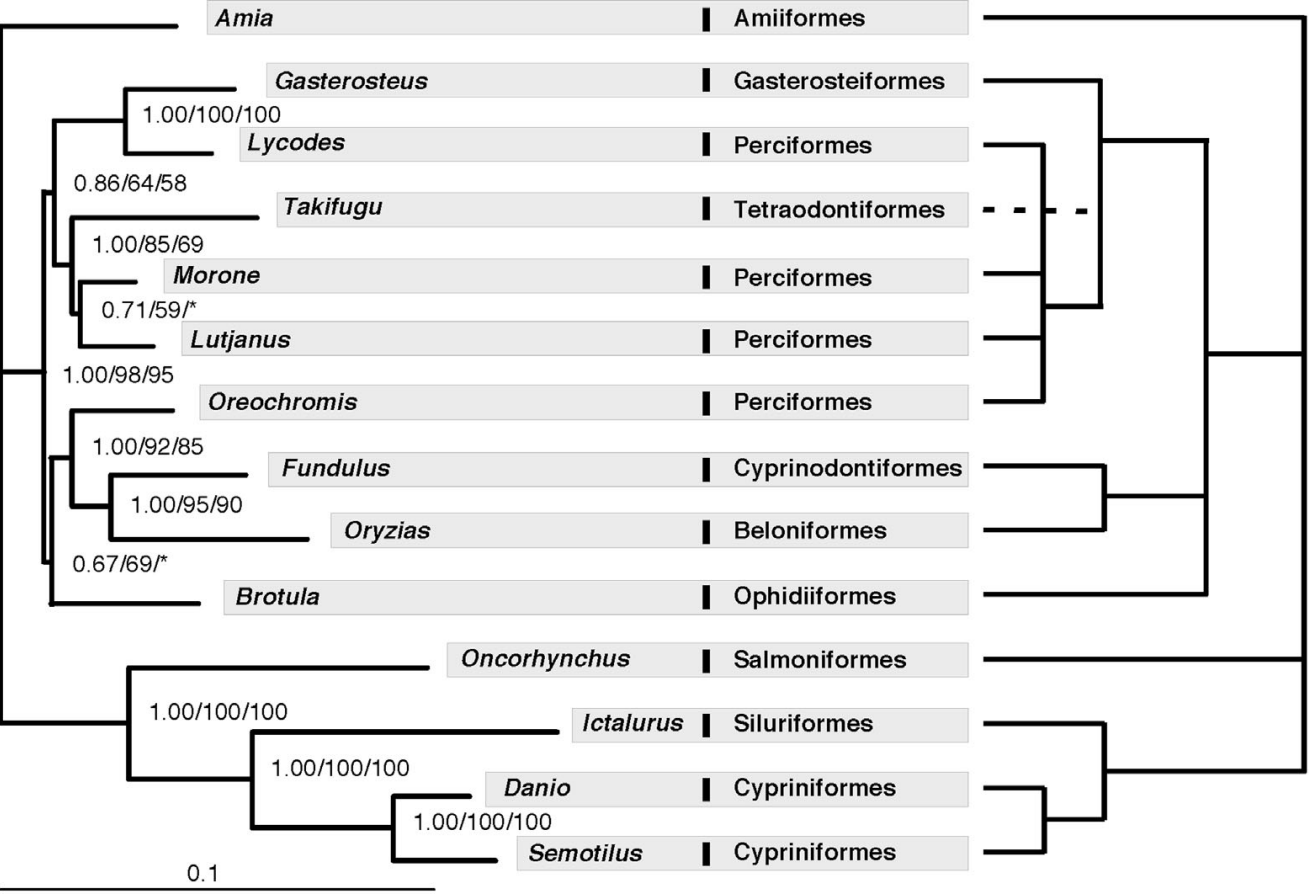
**A comparison of the maximum likelihood phylogram inferred in this study with the conventional phylogeny**. (a) Left panel – the phylogram of 14 taxa inferred from protein sequences of 10 genes; (b) right panel – a "consensus" phylogeny following Nelson [50]. The numbers on the branches are Bayesian posterior probability, ML bootstrap values estimated from protein sequences and ML bootstrap values estimated from RY-coded nucleotide sequence. Asterisks indicate bootstrap supports less than 50.

A phylogeny of the 14 taxa using concatenated sequences of all 10 markers (total of 7,872 bp) was inferred on the basis of protein and DNA sequences. For the protein sequence data, a JTT model with gamma parameter accounting for rate heterogeneity was selected by ProtTest [45]. The data were partitioned by gene, as this strategy was favoured by the Akaike information criterion (AIC) over treating the concatenated sequences as a single partition. Maximum likelihood (ML) and Bayesian analysis (BA) resulted in the same tree (Figure 3a). A similar topology to Figure 3a was obtained by ML analysis of nucleotide sequences with RY-coded nucleotides to address potential artefacts due to base compositional bias [44]. The positions of *Brotula *and *Morone *remain somewhat unresolved, receiving low bootstrap support and conflicting resolution based on either protein or RY-coded nucleotide data. When analyzed separately, all individual gene trees display low support in many branches and none of them has the same topology as the "total evidence" tree based on all 10 genes (see Additional file 3). However, only 6 individual genes exhibit significant differences with the total evidence tree (based on one tailed SH tests with p < 0.05), the exceptions being myh6 (p = 0.113), Gylt (p = 0.091), plagl2 (p = 0.056)), and sreb2 (p = 0.080).

## Discussion

The bioinformatic approach implemented in this study resulted in a large set (154 loci for the zebrafish and torafugu comparison) of candidate genes to infer high-level phylogeny of ray-finned fishes. The actual number of candidate loci depended on the genomes being compared and the fixed search parameters. Experimental tests of a smaller subset (15 loci) demonstrate that a large fraction (2/3) of these candidates are easily amplified by PCR from whole genomic DNA extractions in a vast diversity of fish taxa. The assumption that these loci are represented by a single copy in the fish genomes could not be rejected by the PCR assays in the species tested (all amplifications resulted in a single product), increasing the likelihood that the genetic markers are orthologous and suitable to infer organismal phylogeny. Our method is based on searching, under specific criteria, the available complete genomic databases of organisms closely related to the taxa of interest. Therefore, the same approach that is shown to be successful for fishes could be applied to other groups of organisms for which two or more complete genome sequences exist. Parameter values (L, S, and C) used for the search (Figure 2) may be altered to obtain fragments of different size or with different levels of conservation (i.e., less conserved for phylogenies of more closely related organisms).

An alternative way to develop nuclear gene markers for phylogenetic studies is to construct a cDNA library or sequence several ESTs for a small pilot group of taxa, and then to design specific PCR primers to amplify the orthologous gene copy in all the other taxa of interest [19,46]. The major potential problem with this approach stems from the fact that the method starts with a cDNA library or a set of EST sequences, with no prior knowledge of how many copies a gene has in each genome. As discussed above, this condition may lead to mistaken paralogy. In our approach, we search the genomic database to find single-copy candidates so no duplicate gene copies, if present, would be missed (see below).

Recent studies have proposed whole genome duplication events during vertebrate evolution and also genome duplications restricted to ray-finned fishes [31,32,47,48]. Our results indicate that many single-copy genes still exist in a wide diversity of fish taxa (representing 28 orders of actinopterygian fishes), in agreement with previous estimates that a vast majority of duplicated genes are secondarily lost [34,35]. All 154 candidates were identified as single-copy genes in *D. rerio *and *T. rubripes*, according to our search criteria. Our results also show the 154 candidate genes are randomly distributed in the fish genome (at least among chromosomes of *D. rerio*). In the experimental tests, 10 out of 15 markers were found in single-copy condition in all successful amplifications, including the tetraploid species, *O. mykiss*. However, relaxing the search criteria, and conserving targets less than 50% similar in a subsequent blast search against the zebrafish genome, 7 of the 10 genes were found to have "alignable paralogs" (the 3 exceptions were myh6, tbr1, and Gylt). Genomes of medaka, stickleback, and fugu were also checked for these 3 genes, and no "paralogs" were detected, suggesting the sequences of ray-finned fish collected for these 3 genes are unambiguously orthologous to each other. Phylogenetic analyses for each of the 7 genes that include the putative paralogs found by this procedure produced tree topologies that strongly suggest an ancient duplication event in the vertebrate lineage, before the divergence of tetrapods from ray-finned fishes. Paralogous sequences are placed at the base of the tetrapod-actinopteryigian divergence, or as part of a basal polytomy with the other tetrapod and ray-finned fish sequences. In the terminology proposed by Remm et al. [49] these would be considered out-paralogs. In no case are these sequences nested among ingroup actinopterygian sequences (see Additional file 4), as would be the case expected for in-paralogs [49]. Stringent search critera implemented in our approach followed by phylogenetic analysis can distinguish between orthologs and putative our-paralogs. Although the method will not guarantee that single copy genes amplified by PCR in several taxa are orthologs as opposed to in-paralogs, the existence and identification of genome-scale single-copy nuclear markers should facilitate the construction of the tree of life, even if the evolutionary mechanism responsible for maintaining single-copy genes is poorly known [33].

The molecular evolutionary profiles of the 10 newly developed markers are in the same range as RAG-1, a widely-used gene marker in vertebrates. The genes with high treeness values have intermediate substitution rate, suggesting that optimal rate and base composition stationarity are important factors that determine the suitability of a phylogenetic marker. The phylogeny based on individual markers revealed incongruent phylogenetic signal among 6 of the 10 individual genes. This incongruence suggests that significant biases in the data might obscure the true phylogenetic signal in some individual genes, but the direction of the bias is hardly shared among genes (Additional file 3), justifying the use of genome-scale gene makers to infer organismal phylogeny.

Finally, with respect to the phylogenetic results *per se*, there are two significant areas of discrepancy between the phylogeny obtained in this study (Figure 3a) and a consensus view of fish phylogeny (Figure 3b) [50]. Although these differences could be due to poor taxonomic sampling, we discuss them briefly. First, the traditional tree groups cichlids with other perciforms, whereas our results showed the cichlid *O. niloticus *is more closely related to atherinomorphs (Cyprinodontiformes + Beloniformes) than to other perciforms. This result also was supported by two recent studies analysing multiple nuclear genes [17,51]. The second difference is that the traditional tree groups *Lycodes *with other perciforms, while *Lycodes *was found closely related to *Gasterosteus *(Gasterosteiformes) in our results. Interestingly, the sister-taxa relationship between *Lycodes *and *Gasterosteus *also is supported by recent studies using mitochondrial genome data [38,52]. The difference between our "total evidence" tree and the classical hypothesis is significant based on the new data, as indicated by a one-tailed Shimodaira-Hasegawa (SH) test (p = 0.000) [53].

## Conclusion

We developed a genome-based approach to identify nuclear gene markers for phylogeny inference that are single-copy, contain large exons, and are conserved across extensive taxonomic distances. We show that our approach has practical value through direct experimentation on a representative sample of ray-finned fish, the largest vertebrate clade in need of phylogenetic resolution. The same approach, however, could be applied to other groups of organisms as long as two or more complete genome sequences are available. This research may have important implications for assembling the tree of life.

## Methods

### Genome-scale mining for phylogenetic markers

Whole genomic sequences of *Danio rerio *and *Takifugu rubripes *were retrieved from the ENSEMBL database [54]. Exon sequences with length > 800 bp were then extracted from the genome databases. The exons extracted were compared in two steps: (1) within-genome sequence comparisons and (2) between genome comparisons. The first step is designed to generate a set of single-copy nuclear gene exons (length > 800 bp) within each genome, whereas the second step should identify single-copy, putatively orthologous exons between *D. rerio *and *T. rubripes *(Figure 2). The BLAST algorithm was used for sequence similarity comparison. In addition to the parameters available in the BLAST program, we applied another parameter, coverage (C), to identify global sequence similarity between exons. The coverage was defined as the ratio of total length of locally aligned sequences over the length of query sequence. The similarity (S) was set to S < 50% for within-genome comparison, which means that only genes that have no counterpart more than 50% similar to themselves were kept. The similarity was set to S × > 70% and the coverage was set to C > 30% in cross-genome comparison, which selected genes that are 70% similar and 30% aligned between *D. rerio *and *T. rubripes*. Subsequent comparisons were performed on the newly available genome of stickleback (*Gasterosteus aculeatus*) and Japanese rice fish (*Oryzias latipes*), as described above. We programmed this procedure using PERL programming language to automate the processes and made the source code publicly available on our website [43]. We are in progress to make it available for other genomic sequences and parameter values.

### Experimental testing for candidate markers

PCR and sequencing primers were designed on aligned sequences of *D. rerio *and *T. rubripes *for 15 random selected genes. Primer3 was used to design the primers [55]. Degenerate primers and a nested-PCR design were used to assure the amplification for each gene in most of the taxa. Ten of the 15 genes tested were amplified with single fragment in most of the 36 taxa examined. PCR primers for 10 gene markers are listed in Table 1. The amplified fragments were directly sequenced, without cloning, using the BigDye system (Applied Biosystems). Sequences of the frequently used RAG1 gene were retrieved for the same taxa from GenBank for comparison to the newly developed markers [GenBank: AY430199, NM_131389, U15663, AB120889, DQ492511, AY308767, AF108420, EF033039 – EF033043]. When RAG1 sequences for the same taxa were not available, a taxon of the same family was used, *i.e. Nimbochromis *was used instead of *Oreochromis *and *Neobythites *was used instead of *Brotula*.

### Phylogenetic analysis

Sequences of the 10 new markers in the 14 taxa were used in phylogenetic analysis to assess their performance. Sequences were aligned using ClustalX [56] on the translated protein sequences. Uncorrected genetic distances were calculated using PAUP [57]. Relative substitution rate for each markers were estimated using a Bayesian approach [58]. Relative composition variability (RCV) and treeness were calculated following Phillips and Penny [44]. Prottest [45] was used to chose the best model for protein sequence data and the AIC criteria to determine the scheme of data partitioning. Bayesian analysis implemented in MrBayes v3.1.1 and maximum likelihood analysis implemented in TreeFinder [59] were performed on the protein sequences. One million generation with 4 chains were run for Bayesian analysis and the trees sampled prior to reaching convergence were discarded (as burnin) before computing the consensus tree and posterior probabilities. Two independent runs were used to provide additional confirmation of convergence of posterior probability distribution. Given the biased base composition in the nucleotide data indicated by the RCV value (Table 2), we analyzed the nucleotide data under the RY-coding scheme (C and T = Y, A and G = R), partitioned by gene in TreeFinder, since RY-coded data are less sensitive to base compositional bias [44]. Alternative hypotheses were tested by one-tailed Shimodaira and Hasegawa (SH) test [53] with 1000 RELL bootstrap replicates implemented in TreeFinder.

## Authors' contributions

CL conceived of the study, designed the bioinformatic pipeline, carried out the experimental tests, and drafted the manuscript. GO conceived of the study and helped to draft the manuscript. GZ implemented the bioinformatics pipeline and developed the web pages. GL conceived of the study, designed the bioinformatics pipeline and the web pages, and helped to draft the manuscript. All authors have read and approved the final manuscript.

## Supplementary Material

Additional file 1 Exon ID, exon length, GC content of predicted single nuclear gene markers in zebrafish and torafugu, as well the blast result between orthologous genes. Click here for file

Additional file 2 Results of PCR amplification of 10 new makers in 36 species of ray-finned fishes. Click here for file

Additional file 3 Maximum likelihood phylogeny based on protein sequences of individual genes, zic1, myh6, RYR3, Ptr, tbr1, ENC1, Gylt, SH3PX3, plagl2, and sreb2. Bootstrap value higher than 50% were mapped on branches. Click here for file

Additional file 4 ML phylogenies based on protein sequences of individual genes and their out-paralogs found by relaxing our search criteria to include fragments with similarity < 50%. Click here for file
