# Supplementary material for: A practical approach to phylogenomics: the phylogeny of ray-finned fish (Actinopterygii) as a case study
Source: BMC Evol Biol. 2007 Mar 20;7:44. doi: 10.1186/1471-2148-7-44 (PMC1838417; doi:10.1186/1471-2148-7-44)
Supplement: Additional file 1 — Exon ID, exon length, GC content of predicted single nuclear gene markers in zebrafish and torafugu, as well the blast result between orthologous genes. [file 1471-2148-7-44-S1.rtf]

Supplementary table 1. Exon ID, exon length, GC content of predicted single nuclear gene markers in zebrafish and torafugu, as well the blast result between orthologous genes. 

	Zebrafish		Torafugu		Torafugu vs Zebra‡	
No. of markers	Exon ID	Exon length	GC content		Exon ID	Exon length	GC content		E-value	Identity (%)	
1	ENSDARE00000015655*	968	0.55		SINFRUE00000662228	970	0.57		0	83	
2	ENSDARE00000145053*	1664	0.49		SINFRUE00000786790	1227	0.48		3E-122	83.99	
3	ENSDARE00000117872*	1402	0.49		SINFRUE00000719108	1429	0.57		2E-104	80.06	
4	ENSDARE00000136964*	2605	0.46		SINFRUE00000561510	1483	0.58		3E-101	84.02	
5	ENSDARE00000367269*	1482	0.53		SINFRUE00000681690	1770	0.58		1E-121	79.01	
6	ENSDARE00000465292*	1307	0.48		SINFRUE00000577106	1408	0.53		3E-115	83.13	
7	ENSDARE00000025410*	5811	0.47		SINFRUE00000644156	5799	0.48		2E-91	79.34	
8	ENSDARE00000029022*	2894	0.47		SINFRUE00000628754	1116	0.57		0	86.29	
9	ENSDARE00000039808*	1596	0.49		SINFRUE00000611615	1773	0.57		4E-53	87.26	
10	ENSDARE00000055502*	1745	0.47		SINFRUE00000673034	844	0.58		2E-68	83.47	
11	ENSDARE00000092751†	1636	0.50		SINFRUE00000725450	1636	0.58		5E-49	85.41	
12	ENSDARE00000473520†	946	0.55		SINFRUE00000766736	856	0.56		3E-94	78	
13	ENSDARE00000023056†	948	0.60		SINFRUE00000575639	969	0.61		0	81	
14	ENSDARE00000053911†	534	0.54		SINFRUE00000649188	543	0.56		4E-165	85	
15	ENSDARE00000281285†	640	0.55		SINFRUE00000774212	703	0.57		4E-133	81	
16	ENSDARE00000008379	886	0.48		SINFRUE00000641978	1920	0.53		1E-22	82.32	
17	ENSDARE00000014605	927	0.54		SINFRUE00000776709	1041	0.61		3E-68	88.16	
18	ENSDARE00000021371	2073	0.45		SINFRUE00000577163	1086	0.68		3E-34	83.65	
19	ENSDARE00000025341	971	0.56		SINFRUE00000609687	1935	0.62		5E-28	85.21	
20	ENSDARE00000038832	1100	0.47		SINFRUE00000735032	1056	0.54		9E-93	80.7	
21	ENSDARE00000039062	1188	0.53		SINFRUE00000776320	1203	0.60		2E-38	81.61	
22	ENSDARE00000050276	1216	0.48		SINFRUE00000789399	1089	0.55		6E-23	84.4	
23	ENSDARE00000051716	1867	0.46		SINFRUE00000690882	953	0.55		9E-28	80.49	
24	ENSDARE00000057069	1394	0.48		SINFRUE00000732606	1638	0.50		2E-63	80.68	
25	ENSDARE00000060643	1247	0.49		SINFRUE00000723019	1319	0.49		6E-36	81.36	
26	ENSDARE00000072303	883	0.50		SINFRUE00000618086	844	0.53		2E-53	87.04	
27	ENSDARE00000072794	1940	0.45		SINFRUE00000634400	1263	0.51		2E-60	84.67	
28	ENSDARE00000075160	1203	0.49		SINFRUE00000722545	1206	0.54		2E-26	85.93	
29	ENSDARE00000075532	1002	0.52		SINFRUE00000733528	996	0.59		3E-120	83.43	
30	ENSDARE00000080271	1654	0.46		SINFRUE00000768063	826	0.58		3E-27	82.5	
31	ENSDARE00000083490	846	0.54		SINFRUE00000688050	894	0.53		5E-26	83.53	
32	ENSDARE00000094312	915	0.56		SINFRUE00000626899	948	0.62		4E-30	79.18	
33	ENSDARE00000101104	2013	0.54		SINFRUE00000703687	2533	0.57		2E-25	83.33	
34	ENSDARE00000105670	2202	0.43		SINFRUE00000588080	914	0.62		5E-60	84.24	
35	ENSDARE00000108088	1319	0.49		SINFRUE00000564119	925	0.50		2E-35	80.75	
36	ENSDARE00000111350	948	0.51		SINFRUE00000800129	1022	0.55		1E-30	83.01	
37	ENSDARE00000113193	1994	0.47		SINFRUE00000706470	2496	0.45		4E-29	80.88	
38	ENSDARE00000113527	1290	0.47		SINFRUE00000607191	2304	0.50		4E-32	82.81	
39	ENSDARE00000114437	2899	0.49		SINFRUE00000634453	2307	0.57		1E-25	81.78	
40	ENSDARE00000118208	1545	0.49		SINFRUE00000673962	2670	0.48		7E-34	79.89	
41	ENSDARE00000121572	1446	0.53		SINFRUE00000575529	1126	0.52		5E-33	86.99	
42	ENSDARE00000121853	863	0.57		SINFRUE00000618756	857	0.62		2E-102	86.45	
43	ENSDARE00000127244	1204	0.51		SINFRUE00000699178	1095	0.54		1E-36	84.69	
44	ENSDARE00000135137	1995	0.51		SINFRUE00000646724	2007	0.63		9E-36	79.52	
45	ENSDARE00000140117	888	0.55		SINFRUE00000650663	816	0.56		5E-23	83.03	
46	ENSDARE00000146317	825	0.50		SINFRUE00000623975	1968	0.54		5E-28	81.1	
47	ENSDARE00000149196	1678	0.54		SINFRUE00000648016	924	0.70		2E-22	83.55	
48	ENSDARE00000156722	1054	0.55		SINFRUE00000642853	1655	0.56		3E-32	87.59	
49	ENSDARE00000156742	1647	0.60		SINFRUE00000582617	1570	0.63		4E-28	85.62	
50	ENSDARE00000158301	982	0.54		SINFRUE00000581861	964	0.58		1E-30	81.93	
51	ENSDARE00000158601	1459	0.51		SINFRUE00000663337	904	0.52		7E-44	81.88	
52	ENSDARE00000160152	819	0.55		SINFRUE00000673736	825	0.60		8E-28	81.03	
53	ENSDARE00000164315	840	0.48		SINFRUE00000699740	840	0.51		1E-29	83.51	
54	ENSDARE00000172488	3750	0.43		SINFRUE00000662708	3288	0.57		8E-25	81.69	
55	ENSDARE00000180133	1101	0.52		SINFRUE00000723234	1143	0.53		1E-21	83.23	
56	ENSDARE00000180576	2040	0.40		SINFRUE00000668186	929	0.57		4E-27	84.38	
57	ENSDARE00000182877	2180	0.46		SINFRUE00000652910	1174	0.58		1E-21	84.44	
58	ENSDARE00000189313	891	0.57		SINFRUE00000680694	918	0.55		3E-46	84.75	
59	ENSDARE00000189500	1407	0.43		SINFRUE00000684238	2022	0.46		5E-28	85.62	
60	ENSDARE00000197458	2251	0.51		SINFRUE00000684419	1555	0.54		1E-24	85.94	
61	ENSDARE00000204844	1147	0.51		SINFRUE00000680436	985	0.53		5E-45	82.61	
62	ENSDARE00000206420	1075	0.50		SINFRUE00000572111	1123	0.58		8E-41	85.02	
63	ENSDARE00000206479	1196	0.53		SINFRUE00000580687	1214	0.58		8E-29	85.16	
64	ENSDARE00000219160	1085	0.50		SINFRUE00000607190	1935	0.54		5E-37	82.26	
65	ENSDARE00000219263	1742	0.42		SINFRUE00000666050	1064	0.56		4E-24	82.2	
66	ENSDARE00000229740	1349	0.57		SINFRUE00000690755	1406	0.51		1E-37	80.98	
67	ENSDARE00000254677	2832	0.45		SINFRUE00000575897	1491	0.58		3E-47	82.37	
68	ENSDARE00000264881	954	0.53		SINFRUE00000812202	951	0.55		2E-53	82.3	
69	ENSDARE00000272936	992	0.49		SINFRUE00000699845	1026	0.49		2E-35	81.13	
70	ENSDARE00000281441	2586	0.52		SINFRUE00000610710	2502	0.55		4E-26	86.61	
71	ENSDARE00000281522	802	0.56		SINFRUE00000694569	836	0.56		8E-25	81.73	
72	ENSDARE00000282174	1036	0.52		SINFRUE00000685586	1087	0.64		4E-30	83.78	
73	ENSDARE00000282665	1555	0.47		SINFRUE00000650606	812	0.55		1E-48	83.7	
74	ENSDARE00000285110	1232	0.51		SINFRUE00000627739	1290	0.53		7E-60	83.01	
75	ENSDARE00000285860	2245	0.48		SINFRUE00000623301	882	0.56		2E-34	85.98	
76	ENSDARE00000293219	3252	0.47		SINFRUE00000749920	1509	0.59		1E-27	84.47	
77	ENSDARE00000306073	1548	0.54		SINFRUE00000745372	1551	0.55		1E-21	83.23	
78	ENSDARE00000308452	891	0.55		SINFRUE00000635758	891	0.53		4E-30	80.78	
79	ENSDARE00000311138	1419	0.49		SINFRUE00000599257	1314	0.51		7E-51	80	
80	ENSDARE00000311461	1489	0.55		SINFRUE00000610969	964	0.58		4E-30	83.78	
81	ENSDARE00000323279	1033	0.53		SINFRUE00000601349	1051	0.49		4E-55	81.32	
82	ENSDARE00000332176	1670	0.44		SINFRUE00000602884	1131	0.62		1E-42	80.7	
83	ENSDARE00000335381	829	0.52		SINFRUE00000615205	1020	0.61		2E-99	80.46	
84	ENSDARE00000342020	936	0.61		SINFRUE00000632131	837	0.61		9E-74	82.74	
85	ENSDARE00000344553	854	0.53		SINFRUE00000565494	860	0.58		6E-69	80.89	
86	ENSDARE00000347062	843	0.58		SINFRUE00000601793	1386	0.64		1E-43	81.63	
87	ENSDARE00000358071	833	0.53		SINFRUE00000591640	857	0.62		2E-115	82.93	
88	ENSDARE00000358117	1401	0.49		SINFRUE00000650742	1482	0.51		3E-22	80.51	
89	ENSDARE00000359173	1065	0.49		SINFRUE00000608787	1272	0.56		6E-48	78.6	
90	ENSDARE00000360719	1062	0.58		SINFRUE00000611346	848	0.63		2E-84	81.5	
91	ENSDARE00000360787	1543	0.47		SINFRUE00000667348	992	0.63		1E-33	88.15	
92	ENSDARE00000370814	1439	0.53		SINFRUE00000690230	1430	0.61		5E-55	86.12	
93	ENSDARE00000377477	2762	0.40		SINFRUE00000802706	1055	0.63		3E-46	80.23	
94	ENSDARE00000381363	870	0.60		SINFRUE00000757942	845	0.55		2E-34	91.07	
95	ENSDARE00000386979	2706	0.43		SINFRUE00000592475	1072	0.48		1E-27	83.82	
96	ENSDARE00000389841	868	0.50		SINFRUE00000695948	862	0.60		3E-43	79.43	
97	ENSDARE00000389876	940	0.48		SINFRUE00000695933	1769	0.51		7E-30	85.09	
98	ENSDARE00000391626	1110	0.46		SINFRUE00000695204	1089	0.54		1E-21	83.23	
99	ENSDARE00000392437	818	0.47		SINFRUE00000619713	818	0.57		3E-27	81.7	
100	ENSDARE00000396273	889	0.52		SINFRUE00000656325	1152	0.57		2E-38	82.4	
101	ENSDARE00000397971	887	0.53		SINFRUE00000687744	950	0.59		2E-22	86.21	
102	ENSDARE00000402487	1593	0.47		SINFRUE00000602810	2076	0.43		1E-22	78.12	
103	ENSDARE00000402673	1533	0.53		SINFRUE00000718128	1645	0.49		2E-24	80.56	
104	ENSDARE00000403799	970	0.43		SINFRUE00000597153	854	0.58		2E-25	80.3	
105	ENSDARE00000404770	1797	0.49		SINFRUE00000667654	1947	0.49		8E-30	84.39	
106	ENSDARE00000407314	1174	0.53		SINFRUE00000721499	1122	0.50		1E-98	81.18	
107	ENSDARE00000409838	818	0.53		SINFRUE00000709146	1020	0.49		1E-23	82.42	
108	ENSDARE00000410488	2042	0.51		SINFRUE00000691278	2082	0.54		5E-105	81.26	
109	ENSDARE00000418749	823	0.52		SINFRUE00000730367	919	0.61		4E-33	82.23	
110	ENSDARE00000418930	1156	0.51		SINFRUE00000720787	1175	0.50		3E-34	86.11	
111	ENSDARE00000420489	1653	0.54		SINFRUE00000561462	1590	0.64		1E-40	84.3	
112	ENSDARE00000421998	1027	0.52		SINFRUE00000590718	1030	0.61		2E-72	80.7	
113	ENSDARE00000424213	931	0.54		SINFRUE00000771338	938	0.66		2E-31	83.25	
114	ENSDARE00000429938	831	0.48		SINFRUE00000805544	840	0.50		1E-23	93.24	
115	ENSDARE00000435042	1030	0.55		SINFRUE00000597578	1033	0.65		9E-56	86.84	
116	ENSDARE00000435786	1092	0.53		SINFRUE00000606878	1056	0.55		6E-97	88.79	
117	ENSDARE00000435942	874	0.50		SINFRUE00000717374	874	0.49		8E-93	79.86	
118	ENSDARE00000440228	1767	0.41		SINFRUE00000802590	935	0.49		9E-28	81.9	
119	ENSDARE00000440514	17148	0.41		SINFRUE00000777929	894	0.44		3E-24	83.23	
120	ENSDARE00000441380	924	0.58		SINFRUE00000582889	930	0.58		2E-38	86.29	
121	ENSDARE00000442073	2121	0.45		SINFRUE00000772689	1452	0.48		6E-33	78.6	
122	ENSDARE00000442814	2167	0.44		SINFRUE00000577022	821	0.58		5E-66	82.22	
123	ENSDARE00000452862	822	0.58		SINFRUE00000618557	951	0.66		1E-36	81.09	
124	ENSDARE00000461814	1201	0.52		SINFRUE00000585763	846	0.63		2E-22	84.03	
125	ENSDARE00000463567	2094	0.49		SINFRUE00000669034	1725	0.61		3E-23	84.51	
126	ENSDARE00000468050	2222	0.45		SINFRUE00000565243	1089	0.58		2E-84	81.96	
127	ENSDARE00000472455	862	0.52		SINFRUE00000808658	975	0.58		1E-23	82.94	
128	ENSDARE00000472797	1303	0.43		SINFRUE00000642469	828	0.59		8E-31	84.94	
129	ENSDARE00000479861	927	0.58		SINFRUE00000569048	960	0.60		3E-37	81.31	
130	ENSDARE00000485260	1035	0.56		SINFRUE00000657696	1068	0.52		4E-30	84.85	
131	ENSDARE00000490915	3547	0.47		SINFRUE00000717980	1590	0.53		2E-48	78.73	
132	ENSDARE00000495706	2848	0.47		SINFRUE00000605519	2866	0.51		3E-24	83.54	
133	ENSDARE00000502459	1784	0.45		SINFRUE00000620332	1585	0.55		1E-62	81	
134	ENSDARE00000506413	1323	0.52		SINFRUE00000589030	1032	0.56		6E-23	88.12	
135	ENSDARE00000509406	1212	0.49		SINFRUE00000691757	1104	0.51		6E-57	79.96	
136	ENSDARE00000510312	2289	0.49		SINFRUE00000624350	3542	0.59		6E-32	77.34	
137	ENSDARE00000513536	818	0.61		SINFRUE00000649321	807	0.58		1E-88	83.58	
138	ENSDARE00000513917	3058	0.53		SINFRUE00000784235	3540	0.58		6E-66	89.19	

†markers successfully passed through the in silico as well experimental tests; ‡markers passed through the in silico but failed in the experimental tests; §result of blasting zebrafish sequences with torafugu sequences.
