# Supplementary material for: A practical approach to phylogenomics: the phylogeny of ray-finned fish (Actinopterygii) as a case study
Source: BMC Evol Biol. 2007 Mar 20;7:44. doi: 10.1186/1471-2148-7-44 (PMC1838417; doi:10.1186/1471-2148-7-44)
Supplement: Additional file 2 — Results of PCR amplification of 10 new makers in 36 species of ray-finned fishes. [file 1471-2148-7-44-S2.rtf]

Supplementary table 2. Results of PCR amplification of 10 new makers in 36 species of ray-finned fishes.*
Families	Taxa	Markers	
		zic1	myh6	RYR3	Ptc	tbr1	ENC1	Gylt	SH3PX3	plagl2	sreb2	
Polypteridae	Polypterus congicus	Y	N	N	N	N	N	N	N	N	Y	
Lepisosteidae	Lepisosteus platostomus	Y	Y	N		Partial	N	N	N	N	Y	
Polyodontidae	Polyodon spathula	Y	Y	N	Y	N	N	N	Partial	Y	Y	
Amiidae	Amia calva	Y	Y	Y	Y	Y	Y	Y	Y	Y	Y	
Hiodontidae	Hiodon alosoides	Y	Y	Y	Y	Y	Partial	N	Y	Y	Y	
Elopidae	Elops saurus	Y	Y	N	Y	Y	Partial	N	Y	Partial	Partial	
Clupeidae	Dorosoma cepedianum	Y	Y	Y	Y	Y	N	Y	Y	Y	Y	
Cyprinidae	Danio rerio	Y	Y	Y	Y	Y	Y	Y	Y	Y	Y	
Cyprinidae	Notemigonus crysoleucas	N	Y	Y	Y	Y	Y	Y	Y	Y	Y	
Cyprinidae	Semotilus atromaculatus	Y	Y	Y	Y	Y	Y	Y	Y	Y	Y	
Ictaluridae	Ictalurus punctatus	Y	Y	Y	Y	Y	Y	Y	Y	Y	Y	
Esocidae	Esox lucius	Y	Y	Y	Y	N	Y	N	Y	Y	Y	
Salmonidae	Oncorhynchus mykiss	Y	Y	Y	Y	Y	Y	Y	Y	Y	Y	
Stomiidae	Stomias boa	Y	Y	Y	Y	Y	Y	Y	Y	Y	N	
Synodontidae	Synodus foetens	Y	N	Y	Y	N	Y	Y	Y	Y	Y	
Neoscopelidae	Neoscopelus macrolepidotus	Y	Y	N	Y	Y	Y	Y	Y	Y	Y	
Regalecidae	Regalecus glesne	Y	Y	Y	Y	Y	N	Y	Y	Y	Y	
Aphredoderidae	Aphredoderus sayanus	Y	Y	N	Y	Y	Y	Y	Partial	Partial	Y	
Macrouridae	Coryphaenoides rupestris	N	Y	N	Y	N	Y	N	Y	N	N	
Gadidae	Gadus morhua	Y	Y	Y	Y	N	Y	Y	Y	Y	Partial	
Ophidiidae	Brotula multibarbata	Y	Y	Y	Y	Y	Y	Y	Y	Y	Y	
Batrachoididae	Porichthys plectrodon	Y	Y	N	Y	Y	Y	Y	Y	Y	Y	
Lophiidae	Lophius gastrophysus	Y	Y	Y	Y	Y	Y	N	Y	Y	Y	
Mugilidae	Mugil curema	Y	Y	Y	N	Y	Y	Y	Y	Y	Y	
Poeciliidae	Gambusia affinis	Y	Y	N	Y	Y	Y	Y	Y	Y	Y	
Fundulidae	Fundulus heteroclitus	Y	Y	Y	Y	Y	Y	Y	Y	Y	Y	
Adrianichthyidae	Oryzias latipes	Y	Y	Y	Y	Y	Y	Y	Y	Y	Y	
Atherinopsidae	Labidesthes sicculus	Y	Y	Y	N	Y	Y	N	Y	N	Y	
Gasterosteidae	Gasterosteus  aculeatus	Y	Y	Y	Y	Y	Y	Y	Y	Y	Y	
Holocentridae	Myripristis violacea	Y	Y	Y	Y	Y	Y	Y	Y	N	Y	
Tetradontidae	Takifugu rubripes	Y	Y	Y	Y	Y	Y	Y	Y	Y	Y	
Cichlidae	Oreochromis  niloticus	Y	Y	Y	Y	Y	Y	Y	Y	Y	Y	
Cichlidae	Cichlasoma cyanoguttatum	Y	Y	N	Y	Y	Y	Y	Y	Y	Y	
Moronidae	Morone chrysops	Y	Y	Y	Y	Y	Y	Y	Y	Y	Y	
Lutjanidae	Lutjanus mahogoni	Y	Y	Y	Y	Y	Y	Y	Y	Y	Y	
Zoarcidae	Lycodes terraenovae	Y	Y	Y	Y	Y	Y	Y	Y	Y	Y	
*Markers details are listed in Table 2. Y denotes that a single PCR product was amplified for that taxon, while N indicates that the PCR reaction did not work, and partial indicates weak amplification. Thirty-six species in 33 families and 28 orders were tested. 
