# Supplementary figures and images for: A practical approach to phylogenomics: the phylogeny of ray-finned fish (Actinopterygii) as a case study
Source: BMC Evol Biol. 2007 Mar 20;7:44. doi: 10.1186/1471-2148-7-44 (PMC1838417; doi:10.1186/1471-2148-7-44)

**zic1**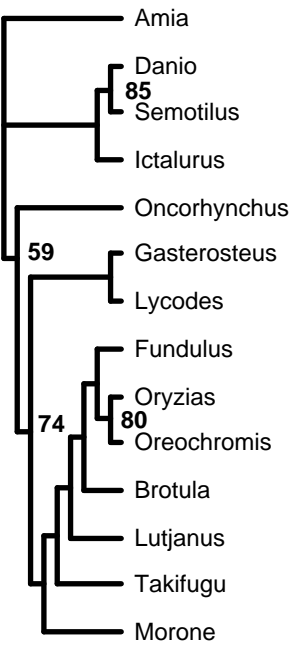**myh6**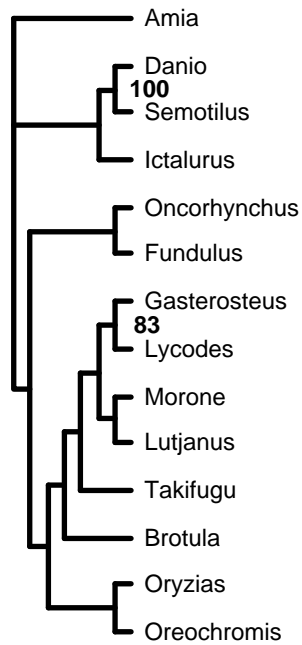**RYR3**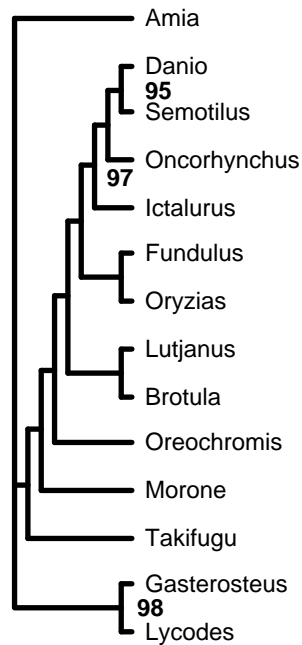**Ptr**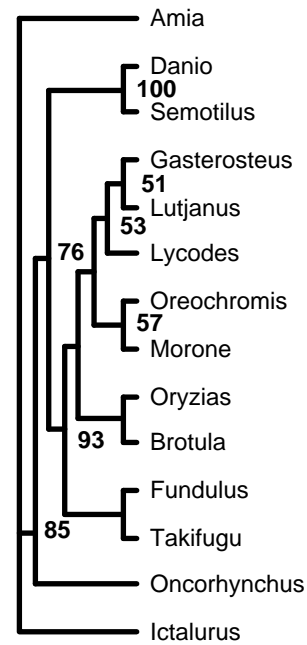**tbr1**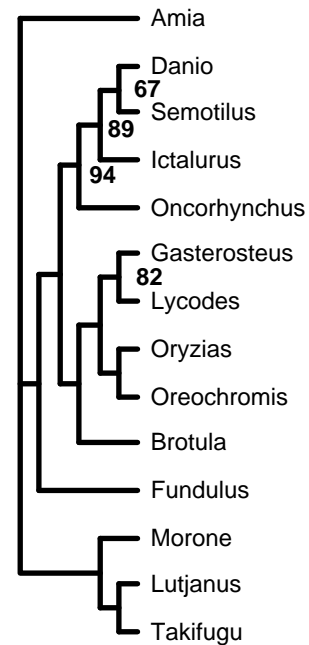**ENC1**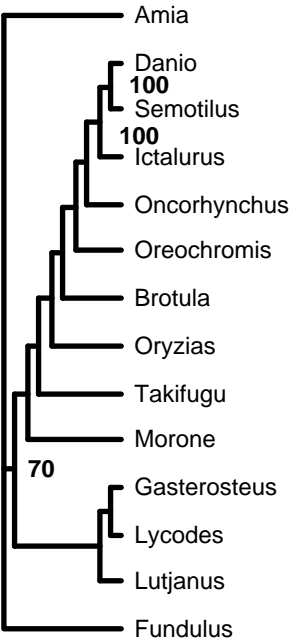**Glyt**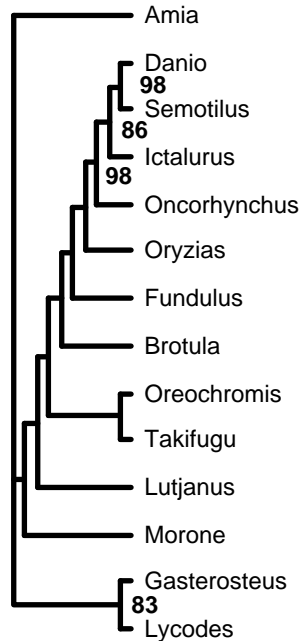**SH3PX3**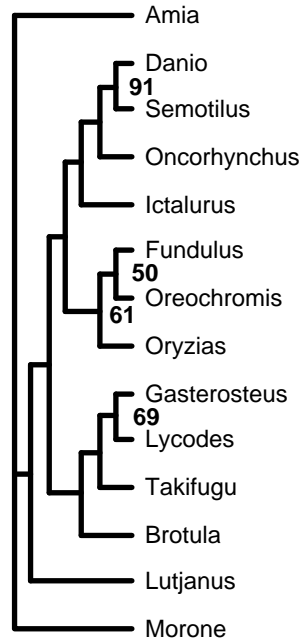**plagl2**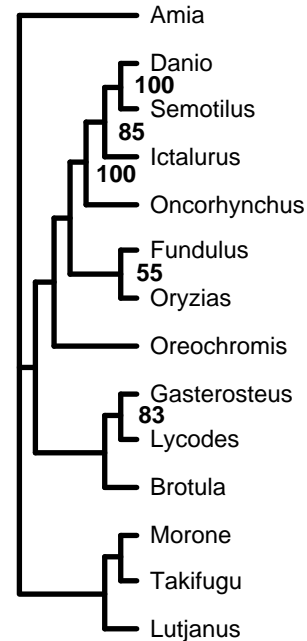**sreb2**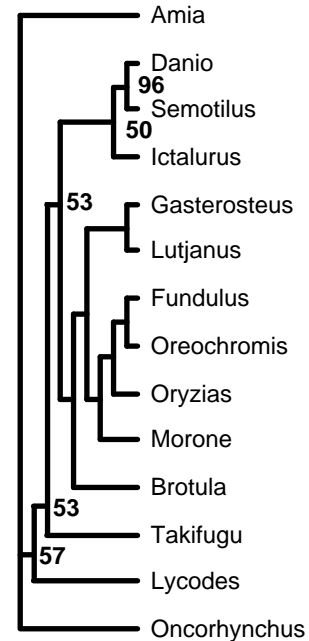

Supplement: Additional file 3 — Maximum likelihood phylogeny based on protein sequences of individual genes, zic1, myh6, RYR3, Ptr, tbr1, ENC1, Gylt, SH3PX3, plagl2, and sreb2. Bootstrap value higher than 50% were mapped on branches. [file 1471-2148-7-44-S3.pdf]

zic1

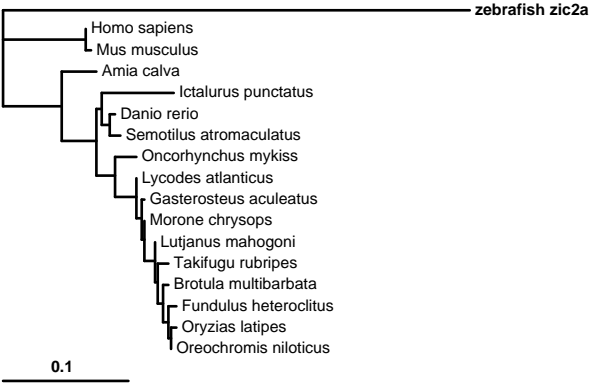

RYR3

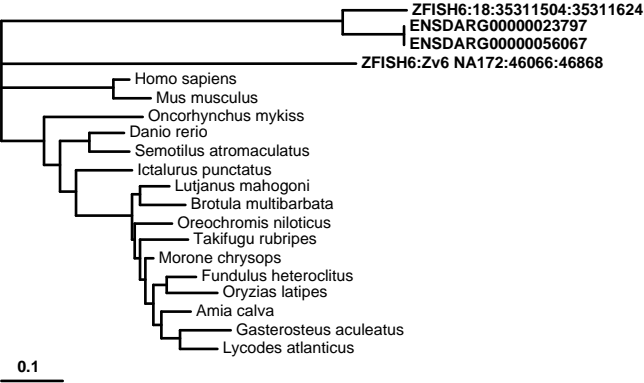

Pt r

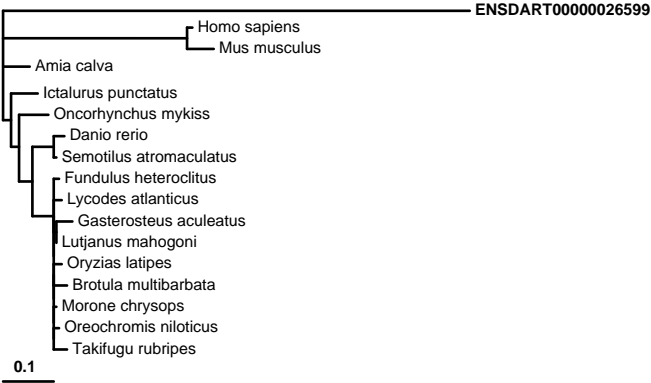

ENC1

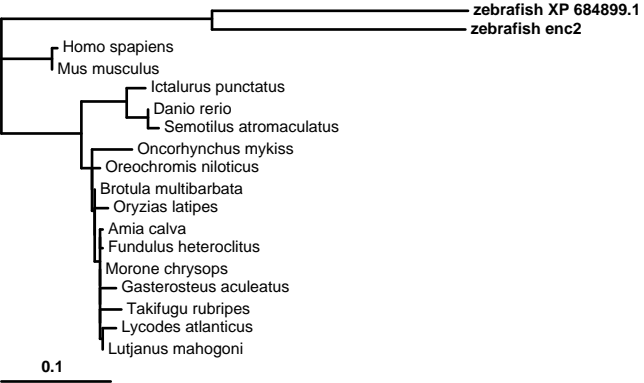

SH3PX3

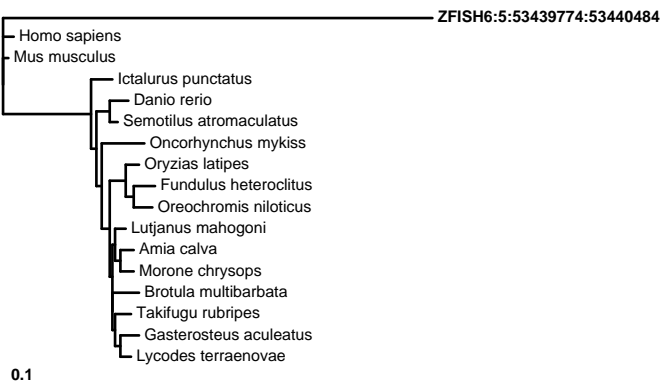

plagl2

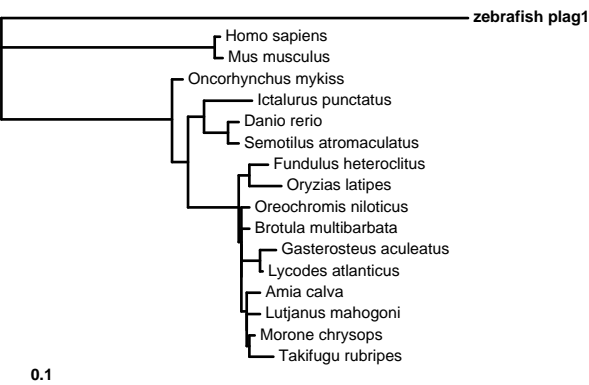

sreb2

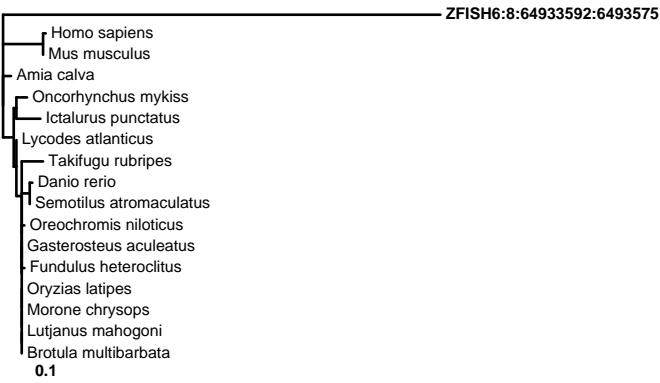

Supplement: Additional file 4 — ML phylogenies based on protein sequences of individual genes and their out-paralogs found by relaxing our search criteria to include fragments with similarity < 50%. [file 1471-2148-7-44-S4.pdf]
